# Supplementary figures and images for: Diversity of plant parasitic nematodes characterized from fields of the French national monitoring programme for the Columbia root-knot nematode
Source: PLoS One. 2022 Mar 8;17(3):e0265070. doi: 10.1371/journal.pone.0265070 (PMC8903304; doi:10.1371/journal.pone.0265070)

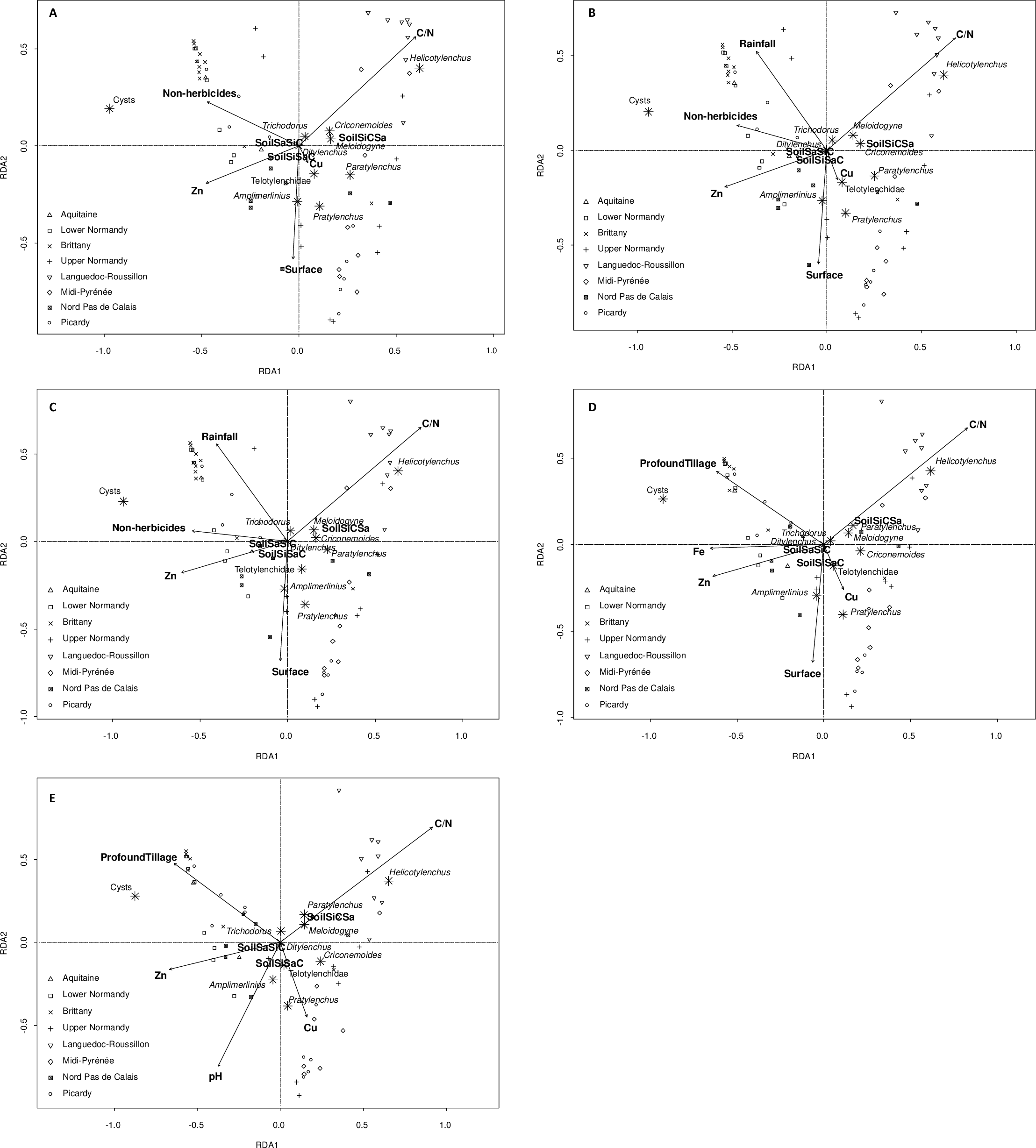

Supplement: S1 Fig — Arrows and bold texts indicate the significant variables after a permutation test. Modalities of soils depend on the proportion of sand, silt and clay in it (SoilSaSiC = majority of sand, then silt then clay; SoilSiCSa = majority of silt, than clay, then sand; SoilSiSaC = majority of silt, then sand, then clay)A: cultivation practices of year n and n-1 are implemented, the first two axes presented explain 38% of the dataset variance; B: cultivation practices of the n to n-2 are implemented, the first two axes presented explain 36% of the dataset variance; C: cultivation practices of year n to n-3 are implemented, the first two axes presented explain 35% of the dataset variance; D: cultivation practices of year n to n-4 are implemented, the first two axes presented explain 39% of the dataset variance; E: cultivation practices of year n to n-5 are implemented, the first two axes presented explain 36% of the dataset variance. (TIF) [file pone.0265070.s001.tif]

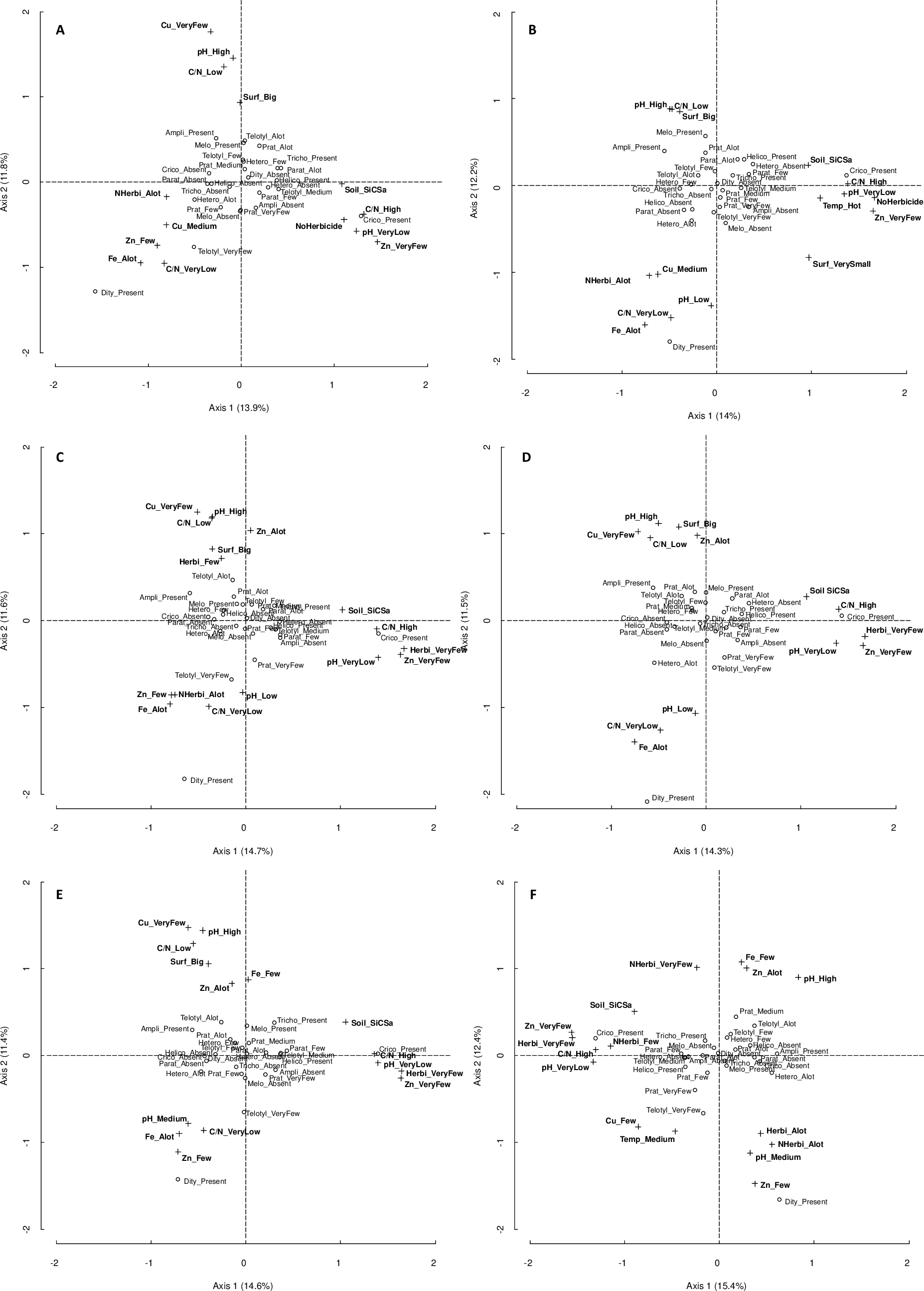

Supplement: S2 Fig — Only the most contributing variables are projected on the maps. PPNs do not contribute to the factorial map construction. Classes for each variable are based on first quartile, median and third quartile. Abbreviations are presented in the supplementary material S2 Table. A: only cultivation practices of year n (year of sampling) are implemented in the analysis; B: cultivation practices of year n and n-1 are implemented; C: cultivation practices of year n to n-2 are implemented; D: cultivation practices of year n to n-3 are implemented; E: cultivation practices of year n to n-4 are implemented; F: cultivation practices of year n to n-5 are implemented. (TIF) [file pone.0265070.s002.tif]
