# Supplementary material for: Diversity of plant parasitic nematodes characterized from fields of the French national monitoring programme for the Columbia root-knot nematode
Source: PLoS One. 2022 Mar 8;17(3):e0265070. doi: 10.1371/journal.pone.0265070 (PMC8903304; doi:10.1371/journal.pone.0265070)
Supplement: Attachment — Submitted filename: PONE-D-21-19128 comments.docx [file pone.0265070.s006.docx]

**Diversity of plant parasitic nematodes characterized from fields of the French national monitoring programme for the Columbia root-knot nematode**

**Short title: Plant parasitic nematodes diversity in French fields**

Nathan Garcia^a,b,^*, Eric Grenier^b^, Alain Buisson^a^, Laurent Folcher^a^

^a^ ANSES, Plant Health Laboratory – Nematology Unit, Domaine de la Motte au Vicomte - 35650 Le Rheu - France

^b^ IGEPP, INRAE, Agrocampus-Ouest, Rennes 1 University, Domaine de la Motte au Vicomte - 35650 Le Rheu - France

* Corresponding author: Nathan Garcia: nathan.garcia@anses.fr

**Abstract**

Plant parasitic nematodes are highly abundant micro-organisms that occur in all agrosystems and some species can have a major impact on crop yields. To avoid the use of chemical agents and to find alternative methods to manage these pests, research studies have mainly focused on plant resistance genes and biocontrol methods involving host plants or natural enemies. A specific alternative method may consist in using non-damaging indigenous species that could compete with damaging non-native species. For this purpose, knowledge about the biodiversity, structure and functioning of these indigenous communities is needed in order to carry out better risk assessments and to develop possible future management strategies. Here, we investigated 35 root crop fields among the French national monitoring program for *Meloidogyne chitwoodi*, in eight regions over two consecutive years. The aims were to describe plant parasitic nematode diversity and to assess the potential effects of anthropic and environmental variables on communities. Community biodiversity included 10 genera and 1 family of plant parasitic nematodes. Despite no significant abundance variations between the two sampling years, structures of communities varied among the different regions. Metadata characterizing the fields and soils, collected over six years from farmers and from a public database, made it possible to evaluate the impact of these variables both on the whole community and on each taxon separately. Our results suggest that, at a large scale, many variables drive the structuration of the communities. Soil variables, whether environmental or anthropic, but also rainfall, explain the population density variations among the geographical areas. The effect of the variables differed among the taxon, but fields with few herbicide applications and being pH neutral with low heavy metal and nitrogen concentrations had higher plant parasitic nematode densities. We discuss how these variables can affect nematode communities either directly or indirectly, by modifying nematode food quality or soil food webs. These types of studies can help both to assess and manage the implantation risk of an introduced nematode and to promote biodiversity and a certain level of abundance of indigenous communities that could compete with the invasive species, without being damaging to crops.

**Introduction**

Plant parasitic nematodes (PPNs) are micro-organisms that feed on plant tissues. A limited number of these nematodes can induce economic losses by decreasing yield or causing visual damage, making the products unsuitable for sale (1). Even though most PPNs are not damaging to crops at natural population levels, some taxa are known to have a major negative impact on crops, even at low abundance. Jones *et al.* (2) listed the 10 most important plant parasitic nematodes, among which *Meloidogyne* spp. (also called root-knot nematodes) are in first place. In particular, the Columbia root-knot nematode, *Meloidogyne chitwoodi,* represents a threat to European agriculture, mainly for root crops (3,4). In order to manage this quarantine pest in fields where it has been detected, several studies have focused on non-host and resistant plants (5,6), or on biocontrol strategies (7). However, studies focusing on preventive strategies, such as preventing the implantation of the damaging nematodes in fields where they are not yet present, are lacking.

The biodiversity of indigenous PPN communities can play a major role in the implantation success of a newly introduced invasive species, since species among these communities could be potential antagonists. For instance, competition between PPN species, but also among communities, has been observed (8–12). While it could be possible to take advantage of such interactions to limit the multiplication rate of *M. chitwoodi* (12), we believe that better knowledge of the diversity and factors affecting the structuration of PPN communities in fields will also be helpful to improve national monitoring programmes and better target the fields that could present a higher risk. In particular, Garcia *et al.* (12) showed, in a greenhouse experiment, that the risk of implantation of *M. chitwoodi* increases in less diverse and less abundant PPN communities. As a result, knowledge about the biodiversity and factors affecting indigenous PPN communities in root crop fields is needed to provide an ecological risk assessment that could be useful to improve the choice of the fields to sample in the framework of the French programme for monitoring the Columbia root-knot nematode.

In agro-ecosystems, that are shaped by frequent anthropic disruptions, the structure and functioning of PPN communities depend mainly on crop management practices (13–18), but also on environmental conditions (17–23). Tillage is probably the most commonly studied cultivation practice and it has been shown that this kind of profound soil work usually has a negative impact on PPN populations (14,24,25). Cultivation practices are known to change the physico-chemical properties of the soil (26–28), which also affects the nematodes in the soil (18,19,21). However, studies often focus on a few environmental conditions or crop practices (15,19,20,29). These studies also take place in restricted areas or with limited experimental designs (13,18,22,23,30). There are, to our knowledge, only a few studies dealing with a larger number of variables and/or at broader spatial scales (31–33). In the present study, we sampled fields that are part of the French monitoring programme for *M. chitwoodi* in several geographical areas in order to (i) describe the biodiversity of PPN communities in fields including a root crop in the crop rotation, (ii) evaluate whether cultivation practices identified in the literature at smaller scales in fact shape PPN communities at larger scale and (iii) assess the potential impact of environmental variables on PPN communities, including climate and physico-chemical conditions of soil. As a consequence, this kind of study could provide knowledge regarding the implantation risk of *M. chitwoodi*, and could help environmental services, in France and other countries, to select the most relevant fields to be sampled for the monitoring programmes. Selection would not only be based on the crops but also on the indigenous communities, especially in terms of densities and biodiversity of the populations observed.

**Results**

**Characterization of the environment and PPN biodiversity**

The sum of monthly precipitation and mean monthly temperatures were collected for the 12 months before each sampling date. Comparison tests showed that the climate was not significantly different over the two sampling years (W = 4.86, *p*-value = 0.283 and W = 638, *p*-value = 0.430 for precipitation and temperature comparisons, respectively).

For the 35 fields sampled in 2015, 10 taxa were identified and counted (9 genera and one family for which we were unable to identify nematodes to the genus level) (Table 1). PPNs identified were *Helicotylenchus*, *Pratylenchus*, *Amplimerlinius*, *Paratylenchus*, *Criconemoides*, *Meloidogyne*, *Ditylenchus*, *Trichodorus*, *Heterodera* and the Telotylenchidae family. Among these taxa, the most frequently found were *Pratylenchus* and Telotylenchidae, both identified in 91% of the fields (Table 1). *Ditylenchus* was found only in 3 fields (Table 1).

| **Table 1: Abundance comparison of the plant parasitic nematode communities found in 2015 and 2016 samplings** | | | | | | | |
| --- | --- | --- | --- | --- | --- | --- | --- |
|  | Mean ± Standard error in 100g of soil | | Comparison 2015-2016 | | | Prevalence (%) | |
|  | 2015 (N=35) | 2016 (N=31) | W | p-value | significance | 2015 | 2016 |
| *Helicotylenchus* | 26.2 ± 9.2 | 67.9 ± 23.0 | 498.0 | 0.537 |  | 46 | 45 |
| *Pratylenchus* | 60.5 ± 10.4 | 67.3 ± 14.6 | 567.0 | 0.757 |  | 91 | 77 |
| *Amplimerlinius* | 14.5 ± 5.8 | 16.5 ± 5.5 | 536.5 | 0.935 |  | 37 | 35 |
| Other Telotylenchidae | 38.7 ± 6.7 | 84.9 ± 29.0 | 380.5 | 0.038 | * | 91 | 97 |
| *Paratylenchus* | 20.9 ± 6.7 | 38.3 ± 13.1 | 481.5 | 0.414 |  | 54 | 55 |
| *Criconemoïdes* | 2.6 ± 1.2 | 5.0 ± 2.6 | 524.0 | 0.746 |  | 20 | 23 |
| *Meloidogyne* | 13.4 ± 5.9 | 8.0 ± 3.0 | 608.0 | 0.353 |  | 49 | 35 |
| *Ditylenchus* | 0.5 ± 0.4 | - | - | - |  | 9 | 0 |
| *Trichodorus* | 1.3 ± 1.3 | 18.6 ± 6.0 | 325.0 | 2.09e^-4^ | *** | 6 | 45 |
| *Heterodera* (juveniles) | 1312.6 ± 637.5 | 1685.6 ± 645.2 | 502.0 | 0.593 |  | 54 | 61 |
| * < 0.05 ; ** < 0.005 ; *** < 0.001 | | | | | | | |

Only nine taxa from among those mentioned above were identified in the 2016 samples from the 31 fields surveyed (Table 1). No additional taxon was found in 2016 compared to 2015, but *Ditylenchus* was no longer found in 2016. *Pratylenchus* and Telotylenchidae were still the most frequently observed PPNs. A comparison between the 2015 and 2016 communities showed a significant difference in *Trichodorus* abundance, increasing from 1.3 ± 1.3 individuals per 100 g of soil in 2015 to 18.6 ± 6.0 in 2016 (W = 325.0, *p*-value = 2.09e^-4^) (Table 1). A significant difference was also observed between abundances of Telotylenchidae (increasing from 38.7 ± 6.7 individuals per 100 g of soil to 84.9 ± 29.0, W = 380.5, *p*-value = 0.038). However, an overall PPN community comparison using the Shannon-Weaver index, showed no significant differences between the 2015 and 2016 PPN communities (W = 733.5, *p*-value = 0.231).

Despite fields were sampled under the scope of the French monitoring program during the two consecutive years, no quarantine species was found in any of the samples and *Meloidogyne* sp*.* mentioned in this article correspond to other species*.*

**Effects of the variables on the whole PPN communities**

A total of 6 transformation-based redundancy analyses (tb-RDAs) were performed, implemented for each analysis the cultivation practices of the previous years, moving backwards (*i.e.* sequential accumulation of the cultural practices over the five years prior to the samplings). Theses analyses aim to assess the effects of anthropic and environmental variables on the overall community and a potential cumulative effect of the cultivation practices over the 5 past years (Fig. 1 and S1). All variables were implemented in each analysis without a preliminary selection step, since tb-RDA enables selection of only the significant variables with a permutation test. To ease the readability of the results, the first tb-RDA (corresponding to the cultural practices of the years of samplings) is presented here (Fig 1) and the others are available in supplementary data (Fig S1).

**Fig. 1 caption: Outputs of the tb-RDA for the cultivation practices of the sampling year.** Arrows and bold text indicate the significant variables after a permutation test. Modalities of soils depend on the proportion of sand, silt and clay in it (SoilSaSiC = majority of sand, then silt then clay; SoilSiCSa = majority of silt, than clay, then sand; SoilSiSaC = majority of silt, then sand, then clay).

The first two axes represented on Fig. 1 explain 31% of the variance of the dataset. Environmental variables (mainly soil properties) appear to be the main drivers of PPN communities. Rainfall, C/N ratio, soil texture and heavy metal concentration (Zn or Cu) are retained on the analysis. The arrow length (indicating the importance of the effect of the variable) of the C/N ratio or rainfalls in particular indicates a key role in structuring of the communities (Fig. 1). For example, *Helicotylenchus* population levels present higher abundance in communities found in soil with a higher C/N ratio. Agricultural practices were not retained on this analysis but cumulative effect of few practices is observed on the following tb-RDA (Fig. S1).

The field surface also appears on the analysis. This result is difficult to explain and could be an artefact. In fact, we were not able to adapt the sampling protocol to the surface sampled, resulting in seven soil cores (regardless the surface), sampled over the longest diagonal of the field. It therefore seems unlikely that the surface may have an impact on PPN communities and we chose to use the surface as a random effect in the following analyses to take this into account.

Many PPN taxa are quite close to the center of the factorial map, indicating that the retained variables, despite a significant impact on overall community structuration, seem to have less importance for each taxon individually. As a consequence, we performed generalized linear mixed models (GLMMs) for each PPN taxon, in order to assess the impact of the same variables on the abundance variation between fields.

**Results of variable selection**

Similarly to the tb-RDA, 6 multiple correspondence analyses (MCAs) were performed, adding the cultivation practices of the past years for each. The first two axes of each factorial map explained around 26% of the dataset variance. For the GLMM, we chose to retain only the variables showing at least two modalities on a minimum of two factorial maps. Hence, soil pH, Zn concentration in the soil, C/N ratio, and number of applications of herbicide products (present on 6, 5, 5 and 2 factorial maps, respectively were retained (factorial maps are available in supplementary data (Fig. S2)).

**Effect of the selected variables**

After the variables selection step, we used a model-averaging approach based on GLMM to test the effective impact of the selected variables on the abundance variation of the PPN taxa. Similarly to the previous analyses, we ran several GLMMs to test a potential cumulative effect of the past applications of herbicide products on the PPN communities. We implemented the four selected variables in the GLMMs. Sixteen first-order models were ranked for each taxon and year or year period. The sum of weight (SW) and 95% confidence interval (CI) were calculated for the variables present in the subset of models with a ΔAICc < 6. A summary of the results is presented in Table 2, while raw detailed results are available in supplementary material (Table S1).

| **Table 2. Equations summarising the model selection approach for each taxa and each year periods. Only significant variables are presented** | | | | | |
| --- | --- | --- | --- | --- | --- |
| Taxon | Year period | Significative variables | Taxon | Year period | Significative variables |
| *Helicotylenchus* | n | Helico ~ C/N - Herbi | *Criconemoides* | n | Crico ~ C/N - Herbi |
|  | n-1 | Helico ~ - Herbi |  | n-1 | Crico ~ - Herbi |
|  | n-2 | Helico ~ C/N |  | n-2 | Crico ~ C/N |
|  | n-3 | Helico ~ - Herbi |  | n-3 | Crico ~ C/N |
|  | n-4 | Helico ~ - Herbi |  | n-4 | Crico ~ C/N |
|  | n-5 | Helico ~ C/N |  | n-5 | Crico ~ C/N |
| *Pratylenchus* | n | Prat ~ - C/N - Zn | *Meloidogyne* | n | Melo ~ C/N + pH - Zn |
|  | n-1 | Prat ~ - C/N - Zn - Herbi |  | n-1 | Melo ~ C/N + pH - Zn |
|  | n-2 | Prat ~ - C/N - Zn - Herbi |  | n-2 | Melo ~ C/N + pH - Zn |
|  | n-3 | Prat ~ - C/N - Zn - Herbi |  | n-3 | Melo ~ C/N + pH - Zn |
|  | n-4 | Prat ~ - C/N - Zn - Herbi |  | n-4 | Melo ~ C/N + pH - Zn |
|  | n-5 | Prat ~ - C/N - Herbi |  | n-5 | Melo ~ C/N + pH - Zn |
| Telotylenchidae | n | Telotyl ~ pH - Zn - Herbi | *Trichodorus* | n | Tricho ~ - Zn - Herbi |
|  | n-1 | Telotyl ~ pH - Zn + Herbi |  | n-1 | Tricho ~ - Zn |
|  | n-2 | Telotyl ~ pH - Zn + Herbi |  | n-2 | Tricho ~ - Zn - Herbi |
|  | n-3 | Telotyl ~ pH - Zn + Herbi |  | n-3 | Tricho ~ - Zn |
|  | n-4 | Telotyl ~ pH - Zn + Herbi |  | n-4 | Tricho ~ - Zn - Herbi |
|  | n-5 | Telotyl ~ - Herbi |  | n-5 | Tricho ~ - Zn |
| *Paratylenchus* | n | Parat ~ C/N + pH | *Heterodera* | n | Hetero ~ Zn - Herbi |
|  | n-1 | Parat ~ C/N + pH |  | n-1 | Hetero ~ Zn |
|  | n-2 | Parat ~ C/N + pH |  | n-2 | Hetero ~ Zn - Herbi |
|  | n-3 | Parat ~ C/N + pH |  | n-3 | Hetero ~ Zn - Herbi |
|  | n-4 | Parat ~ C/N + pH |  | n-4 | Hetero ~ - Herbi |
|  | n-5 | Parat ~ - Herbi |  | n-5 | Hetero ~ Herbi |
| No variables had a significant impact on *Amplimerlinus* population variations, explaining its absence in this table. | | | | | |

None of these variables impacted *Amplimerlinius* abundance. One to three out of the four retained variables had a significant impact on PPN abundance, depending on the taxa and the time period considered. Soil pH – varying between 5.72 and 7.89 in our dataset – had a positive effect on the abundance of *Meloidogyne*, *Paratylenchus* and the Telotylenchidae. Zn concentration in the soil – ranging from 1.23 to 11.12 mg/kg – had a negative impact on the abundance of *Trichodorus, Meloidogyne*, *Telotylenchidae* and *Pratylenchus*, for which the population level seemed to decrease in higher Zn concentration soils. However, Zn concentration had a positive impact on the abundance of *Heterodera*. Similarly, C/N ratio – ranging from 7.28 to 25.87 – had a positive impact on *Meloidogyne, Criconemoides, Paratylenchus* and *Helicotylenchus*, but a negative impact on *Pratylenchus* abundance. The use of herbicides, the only cultivation practice implemented in the GLMM following the MCA selection step, had a negative effect in the short or long term, depending on the PPN. PPN population levels were lower in fields where herbicide products were often used. The results are unclear concerning the Telotylenchidae as herbicides had a negative impact if the sampling year (short-term) or the 5 years before the samplings (longest-term) are considered. However, herbicides had a positive impact on Telotylenchidae if cultivation practices of the years before the samplings, and 2 to 4 years before the sampling are considered.

**Discussion**

**PPN community biodiversity**

This study describes the PPN communities in agrosystems including root crops in France. Sampling in various geographical areas highlighted differences in biodiversity between communities due to environmental heterogeneity and various crop management approaches. The overall biodiversity, with 10 PPN taxa identified across the fields, is close to that of other similar agrosystems studied in the literature (33–37). The fields were chosen among those monitored as part of the French monitoring programme for the Columbia root-knot nematode, based on 2014 or 2015 crops (root vegetable crops), without information about crop rotation. It appears on the basis of crop management information collected from farmers that root crops were not very frequent in crop rotations, and farmers mainly grew wheat or maize, regardless of the region. The majority of the taxa were present in several regions but their abundance seems to vary. The presence of the same PPN taxa in several regions could be due to their generalist feeding habits at the genus level and their plasticity regarding environmental conditions. Identified PPN taxa are mainly colonisers and are not particularly sensitive to environmental stress, explaining their presence in the majority of the regions (38–40). However, one of the limitations here is that despite the large geographic scale investigated, it was not possible to sample numerous fields in each region. It is therefore not possible to statistically compare the communities between regions more deeply. Interestingly, despite homogeneity between 2015 and 2016 PPN communities, *Trichodorus* was the only genus substantially more abundant in 2016. This nematode feeds ectoparasitically mainly on sugar beet and carrot, and can induce economic issues (1,41). One of these two crops was grown in 2015 in the fields with high 2016 abundance of *Trichodorus*, whereas crops cultivated before 2015 were generally cereals. The presence of a suitable host plant could explain this increase in abundance. Furthermore, *Trichodorus* has a very large host range (42), including many weeds that could have been present in sampled fields despite the use of herbicide chemicals. Similarly, the significant increase in Telotylenchidae between the two sampling years could be due to the presence of a better host plant in 2016. The other PPN taxa were not significantly different between the two years, probably because of short crop rotations dominated by cereals.

**The effects of variables on the overall PPN communities**

Abiotic factors, mainly soil conditions, are known to be important factors for PPNs and nematodes in general (21,43). Heavy metals such as Zn or Cu can shape PPN communities because nematodes can be more or less susceptible to these substances (19). In fact PPN abundance has been shown to be negatively correlated to Zn concentrations. The toxicity of heavy metals has been studied in *Caenorhabditis elegans*, a free-living nematode, and high concentrations have been shown to drastically increase mortality in both adult and juvenile stages (44,45), but also to reduce the growth of the nematodes, particularly in the descendants of intoxicated parents (45). Heavy metals also alter the movement behaviour of the intoxicated individuals, possibly by attacking the muscles and the neural network (45). At the community level, little information is available about PPNs but heavy metals have a strong impact on free-living soil nematode communities (46,47). Consequently, nematodes have developed a broad range of behavioural and physiological mechanisms (summarised in Ekschmitt and Korthals (19)) enabling avoidance, tolerance or detoxification of various heavy metals. The effects of heavy metals can also be indirect through reduced plant growth (48) and thus lower quality nutritional content for PPNs, as these organisms depend on their host plants for nutrition.

Soil texture have also been shown to have a major impact on PPN community structuration (18,21,49). Sand, clay and silt proportions may affect the water retention but also the amount of minerals and organic matter present in soils (21,50). This will impact plant growth and thus food quality for PPNs, but also movements through the water film around the soil particles, eased by the retention of root exudates (51).

Obviously, climate was a significant variable affecting PPN communities studied here, especially rainfall. Even at a larger scale (worldwide) Nielsen *et al.* (52) also observed an impact of rainfall on PPN communities. According to their observations, rainfall seems to be the main factor affecting nematode communities in general (including non-PPNs), and temperature seems to be more important for PPNs in particular, whereas soil variables were not important (52). It is possible that in our study, soil variables were more important than climate compared to a worldwide scale, as France is not a very large country and climate variations are relatively low, despite north to south variations (53). Precipitation, and thus moisture, increases plant growth, facilitates PPN movement in soil (54), and favours the PPN life cycle, for instance by increasing egg hatching (55).

Certain cultivation practices (e.g. soil work and use of non-herbicide products) also seem to play a key role in the structuration of the PPN communities in the fields, even though these practices did not impact the abundance of the PPN taxa individually. Tillage, for example, may impact food accessibility for PPNs, as well as their living habitats, mainly their living depth (56,57). Furthermore, some PPNs are less sensitive to environmental changes as reported for *Paratylenchus* (38) and thus may benefit from the soil disturbance compared to the other PPNs. Non-herbicide plant protection products (*i.e.* insecticides, fungicides or molluscicides) may eliminate potential natural enemies of PPNs such as nematophagous fungi (58), but they also have a negative impact on non-target organisms such as the PPNs (59), explaining their structuring role on PPN communities.

**Impact of the variables on each PPN taxon**

Effects of the anthropic and environmental variables were also tested separately on the different PPN taxa using GLMMs. In our data, crop rotations were not retained in the analyses, while crops are known to be important for PPN community structure (60–62). The results here potentially mask the effects of the crops since, at this large geographic scale, environmental conditions (soil and climate) are much more diverse than the cultivation practices between the regions. Despite the presence of a root crop in the rotations, the dominance of cereals in all sampled fields, regardless of the region, probably homogenised the PPN communities. Additionally, it is possible that the effect of crop rotations was masked and would have required more contrasted crop rotations to be highlighted.

We observed that herbicides have a significant negative impact, at least on long-term use, on the abundance of several PPN taxa (*Pratylenchus*, *Trichodorus*, *Helicotylenchus*, *Paratylenchus*, *Criconemoides*, *Heterodera* or Telotylenchidae), as has been reported previously (33,63,64). It is not possible to rule out a potential direct effect of the products depending on the dose used (65), but it is more likely to be an indirect effect by removal of potential host weeds. Positive effects of herbicides can be observed on Telotylenchidae for certain groups of years, possibly because of a negative impact on the other PPNs. Unidentified species among this family could be more competitive on the few remaining weeds. However, this interpretation is speculative, as the weed compositions, as well as potential intercrops between each year, were not assessed here.

In this study, soil physico-chemical variables (pH, soil C/N ratio and Zn) seems to be the main drivers of the PPN abundance variations for the identified taxa. Their effects appeared to be positive or negative, probably depending on the PPN ecological niche. The C/N ratio reflects organic matter and nitrogen enrichment. These variables generally characterise inputs to increase yields. As such, a positive effect is probably due to better quality food sources for PPNs. However, high nitrogen inputs for example have also been shown to be deleterious for soil nematodes because of the nematicidal effect of ammonia (66,67). This seems to be observed here because in the fields sampled for this study, the C/N ratios appear to be relatively low (<10) – except in the four fields sampled in Languedoc-Roussillon – and PPN population levels appear to be lower in soil with lower C/N ratios. With our dataset, Zn appears to negatively impact the abundance of *Trichodorus, Meloidogyne, Pratylenchus* and Telotylenchidae, confirming the results observed with the tb-RDA analyses for the whole PPN community.

**Conclusions**

Based on our results at the spatial scale of several regions of France, PPN community diversity does not seem to be directly affected by crop rotations, whereas this is known to be an important factor for PPN community structure at local or even landscape scales (13,18,30,33). However, among the anthropic activities, pesticide use and soil work had a greater impact than the crop rotations in the fields sampled. Here, it appears that soil and climate conditions are more important than cultivation practices for PPN population levels, and these conditions may mask the effects of certain cultivation practices. Heavy metals, soil pH, and rainfall deeply modify PPN ecosystems, affecting their environment and access to - or quality of - food resources. We showed, with 35 sampled fields in eight different geographical regions, that local climate and soil variations, but also certain cultivation practices, could explain the PPN community variations, despite similar diversity. However, it should be emphasized that anthropic activities, mainly in agrosystems, completely change the soil conditions, with high inputs, or soil work, which directly or indirectly have an impact on PPNs.

Even in the similar agrosystems sampled here, PPN communities may vary and it is probable that the differences in diversity would be higher in more different crop rotations. In this case, we can hypothesise that PPN communities in the different agrosystems would not have an equal resilience capacity to introduction of a new species, such as *Meloidogyne chitwoodi*, and an equal capacity to limit its development. It has been shown previously in greenhouse experiments that more diverse communities, especially those with high overall abundance of PPNs, regardless of the species, are able to limit the establishment of *M. chitwoodi* (12). Given these results, it seems important to consider herbicide use, nitrogen inputs, and soil physico-chemical composition, as well as the cultivated crop, to select the fields sampled for the French national monitoring programme for the Columbia root-knot nematode. Further investigations are also needed to assess the potential of the different natural communities, which here do not damage crops, to control and limit the development of frequently introduced species, with the aim of proposing alternatives to pesticide use.

**Methods**

**Ethics statement**

No specific permits were required for the samplings. Permissions for sampling the fields were granted by the farmers (landowners). Furthermore, the 35 fields sampled in this study were also part of the 2015 French national monitoring programme for the Columbia root-knot nematode. The lands are not protected in any way and no protected plant or animal was sampled.

**Sampling and characteristics of the studied areas**

Sampling was carried out from June to September 2015 in the same fields as those sampled in the framework of the national monitoring programme for quarantine nematodes. Eight regions were investigated: Aquitaine, Brittany, Languedoc-Roussillon, Midi-Pyrénées, Nord-Pas de Calais, Upper and Lower Normandy and Picardy. Sampling was repeated in 2016 in the same fields when possible. All these fields are present in geographical areas defined as temperate regions with warm summers (53). Mean monthly temperatures and rainfall data were collected from an agricultural data website (68) monitoring climate in fields in France, for each sampling over the year before the sampling date.

A total of 35 fields, ranging from 0.13 ha to 6.64 ha and representing 31 farms were sampled in 2015, and 31 of them were sampled again in 2016 as farmers from the other four fields (3 fields from Brittany and 1 field from Nord-Pas de Calais) did not complete the survey. For each field, information about cultivation practices from 2010 to 2016 was collected from farmers through a survey: crop rotations, type and number of soil works (profound tillage at 30 cm or deeper and superficial work at less than 30 cm deep), and number of applications of plant protection products (herbicides or non-herbicides (including fungicides, insecticides and molluscicides). The physico-chemical properties of soils, including pH, organic C/N ratio, soil texture and heavy metal quantities, were obtained from a public database of the National Institute of Agronomical Research (69). Soil samples (around 1.5 kg) were composed of seven elementary soil cores taken at 30 cm depth along the longest diagonal of each field with a manual auger (diameter 2.5 cm). The GPS coordinates were recorded at each of the seven sample points in 2015 in order to repeat the same sampling in 2016.

**Nematode extraction and identification**

The extraction protocol was the same as in Garcia *et al.* (33). Briefly, free-living and cyst nematodes were extracted separately. We used an Oostenbrink elutriator (Meku, Germany) followed by two centrifugations (Hettich Rotanta 460, Germany) to purify the extract for free-living nematodes. PPN genera were identified and counted, after a standardisation step (dilution of the soil extract), based on morphological criteria using a binocular magnifier or a microscope when more precision was needed (70–72). Cysts were manually isolated under magnifier binoculars after filtration on two successive thieves. Genera were identified based on cyst morphology. Furthermore, the number of cysts was transformed into the number of larvae per cyst, through extrapolation based on personal observations, to enable abundance comparisons with the other PPN taxa.

**Statistical analysis**

For this study, we considered 15 different variables, both quantitative and qualitative: year of sampling, mean monthly rainfall, mean monthly temperature, C/N ratio, soil texture, soil Cu, Zn and Fe concentrations, soil pH, field surface, crop rotation, number of superficial soil works, number of profound tillage, number of herbicide and non-herbicide products used.

All statistical analyses were conducted using R software (73). A Wilcoxon test was used to assess differences between PPN taxa abundance in 2015 and 2016.

As samples were taken in quite different areas (mainly regarding environmental conditions), we wanted to assess the impact of both cultivation and environmental variables on the whole PPN community. Transformation-based redundancy analysis (tb-RDA) was performed using the “Vegan” R-package (74). Since our dataset contained many zeros and according to Legendre and Gallagher (2001) (75) and Legendre and Legendre (2012) (76), we transformed our community data using Hellinger transformation (77). Furthermore, the significance of the most contributive variables, was tested according to the Akaike information criterion (AIC) after both forward and backward selection with permutation tests. We performed six tb-RDAs to assess the impact of the past cultivation practices on the present PPN communities. For each analysis, the cultivation practices of the previous years were implemented one by one, moving backward (e.g*.* practices of year n for the first tb-RDA, practices of years n and n-1 for the second, practices of the years n, n-1 and n-2 for the third *etc.*), in order to evaluate whether accumulation of soil work or pesticides over the years could reveal a cumulative effect.

The effects of the variables on the abundance variation of each PPN taxon were evaluated, using a statistical strategy similar to that described by Garcia *et al.* (33). Briefly, we first used MCA using the “FactoMineR” R-package (78) to describe our dataset and to select the main contributing variables without any a priori knowledge (79,80). MCA enables implementation of both qualitative and quantitative variables after grouping the values of the quantitative variables into two to four classes, in a way as balanced as possible to avoid giving greater weight to one modality compared to the others. Limits of each classes were the first and third quartiles and the median of the distribution of each variable. The PPN abundances were considered as supplementary variables and only the most contributive classes or modalities of variables (with an absolute contribution higher than twice the mean absolute contribution (81,82)) were kept on the factorial maps. Six MCA analyses were also performed, adding for each, the cultivation practices of the past years similarly to the tb-RDAs. We retained the variables for which two modalities at least were present on not less than two factorial maps. Then, the effective relationships between these selected variables and the abundance of the PPN taxa were tested using a model-averaging approach through the corrected Akaike information criterion (AICc) (79,80) on generalised linear mixed Poisson models (GLMMs) (83). We used the “lme4” R-package (84) and the “MuMIn” R-package (85). We considered the field surface as a random variable since the sampling protocol was not adapted according to the surface. According to Grueber *et al.* (80), we transformed our explanatory variables using Gelman's (2008) (86) approach, using the “arm” R-package (87), when the models failed to converge. The SW and 95% CI were calculated for the variables present in the subset of models with a ΔAICc < 6. Furthermore, if the 95% CIs included zero, the effect of the variables was considered uncertain (80) and thus not kept for the final model.

**Acknowledgments**

We would like to thank all the farmers who allowed us to sample PPNs in their fields and the French Regional Services for Food for their help contacting the farmers. Thanks are also due to all the colleagues who gave their time to help sample, identify and count PPNs and discuss the results: Valentin Boulenger, Alain Buisson, Magali Esquibet, Sylvain Fournet, Didier Fouville, Josselin Montarry, Christophe Piriou, Catherine Porte, Elsa Ruliat, and Corinne Sarniguet.

**References**

1.	Bridge J, Starr JL. Plant nematodes of agricultural importance: A Colour Handbook. CRC Press; 2007. 154 p.

2.	Jones JT, Haegeman A, Danchin EGJ, Gaur HS, Helder J, Jones MGK, et al. Top 10 plant-parasitic nematodes in molecular plant pathology. Molecular Plant Pathology. 2013 1;14(9):946–61.

3.	Trudgill DL. Parthenogenetic root-knot nematodes (*Meloidogyne* spp.); how can these biotrophic endoparasites have such an enormous host range? Plant Pathology. 1997 1;46(1):26–32.

4.	Trudgill DL, Blok and VC. Apomictic, polyphagous root-knot nematodes: exceptionally successful and damaging biotrophic root pathogens. Annual Review of Phytopathology. 2001;39(1):53–77.

5.	Jensen KB, Griffin GD. Resistance of auto- and allotetraploid triticeae species and accessions to *Meloidogyne chitwoodi* based on genome composition. J Nematol. 1997;29(1):104–11.

6.	van der Beek JG, Poleij LM, Zijlstra C, Janssen R, Janssen GJW. Variation in virulence within *Meloidogyne chitwoodi*, *M. fallax*, and *M. hapla* on *Solanum* spp. Phytopathology. 1998 1;88(7):658–65.

7.	Wishart J, Blok VC, Phillips M, Davies KS. *Pasteuria penetrans* and *P. nishizawae* attachment to *Meloidogyne chitwoodi, M. fallax* and *M. hapla*. Nematology. 2004;6(4):507–10.

8.	Lasserre F, Rivoal R, Cook R. Interactions between *Heterodera avenae* and *Pratylenchus neglectus* on wheat. J Nematol. 1994;26(3):336–44.

9.	Umesh KC, Ferris H, Bayer DE. Competition between the plant-parasitic nematodes *Pratylenchus neglectus* and *Meloidogyne chitwoodi.* J Nematol. 1994;26(3):286–95.

10.	Villenave C, Cadet P. Interactions of *Helicotylenchus dihystera*, *Pratylenchus pseudopratensis*, and *Tylenchorhynchus gladiolatus* on two plants from the Soudano-Sahelian zone of West Africa. Nematropica. 1998;28(1):31–9.

11.	Diez A, Lawrence GW, Lawrence KS. Competition of *Meloidogyne incognita* and *Rotylenchulus reniformis* on cotton following separate and concomitant inoculations. J Nematol. 2003;35(4):422–9.

12.	Garcia N, Grenier E, Sarniguet C, Buisson A, Ollivier F, Folcher L. Impact of native plant-parasitic nematode communities on the establishment of *Meloidogyne chitwoodi*. Plant Pathology. 2018;67(9):2019–27.

13.	Parmelee RW, Alston DG. Nematode trophic structure in conventional and no-tillage agroecosystems. J Nematol. 1986;18(3):403–7.

14.	Freckman DW, Ettema CH. Assessing nematode communities in agroecosystems of varying human intervention. Agriculture, Ecosystems & Environment. 1993;45(3–4):239–61.

15.	Neher DA. Nematode communities in organically and conventionally managed agricultural soils. J Nematol. 1999;31(2):142–54.

16.	Griffiths BS, Daniell TJ, Donn S, Neilson R. Bioindication potential of using molecular characterisation of the nematode community: response to soil tillage. European Journal of Soil Biology. 2012;49:92–7.

17.	Villenave C, Jimenez A, Guernion M, Pérès G, Cluzea D, Mateille T, et al. Nematodes for soil quality monitoring: results from the RMQS BioDiv Programme. Open Journal of Soil Science. 2013;03(01):30–45.

18.	Palomares-Rius JE, Castillo P, Montes-Borrego M, Navas-Cortés JA, Landa BB. Soil properties and olive cultivar determine the structure and diversity of plant-parasitic nematode communities infesting olive orchards soils in southern Spain. PLoS ONE. 2015;10(1):e0116890.

19.	Ekschmitt K, Korthals GW. Nematodes as sentinels of heavy metals and organic toxicants in the soil. J Nematol. 2006;38(1):13–9.

20.	Wei C, Zheng H, Li Q, Lü X, Yu Q, Zhang H, et al. Nitrogen addition regulates soil nematode community composition through ammonium suppression. PLoS ONE. 2012;7(8):e43384.

21.	Mateille T, Tavoillot J, Martiny B, Fargette M. Importance of soil characteristics for plant-parasitic nematode communities in European coastal foredunes. European Journal of Soil Biology. 2014;64:53–60.

22.	Griffiths BS, Bengough AG, Neilson R, Trudgill DL. The extent to which nematode communities are affected by soil factors-a pot experiment. Nematology. 2002;4(8):943–52.

23.	Griffiths B, Neilson R, Bengough AG. Soil factors determined nematode community composition in a two year pot experiment. Nematology. 2003;5(6):889–97.

24.	Pankaj SH, Gaur HS, Singh AK. Effect of zero tillage on the nematode fauna in a rice-wheat cropping system. Nematol Medit. 2006;34:175–8.

25.	Stirling GR, Halpin NV, Bell MJ, Moody PW. Impact of tillage and residues from rotation crops on the nematode community in soil and surface mulch during the following sugarcane crop. International Sugar Journal. 2011;113(1345):56–64.

26.	Shipitalo MJ, Protz R. Comparison of morphology and porosity of a soil under conventional and zero tillage. Canadian Journal of Soil Science. 1987;67(3):445–56.

27.	Dick RP. A review: long-term effects of agricultural systems on soil biochemical and microbial parameters. Agriculture, Ecosystems & Environment. 1992;40(1):25–36.

28.	Duplay J, Semhi K, Errais E, Imfeld G, Babcsanyi I, Perrone T. Copper, zinc, lead and cadmium bioavailability and retention in vineyard soils (Rouffach, France): the impact of cultural practices. Geoderma. 2014;230–231:318–28.

29.	Li X, Ding C, Liu J, Zhang T, Wang X. Evident response of the soil nematode community to consecutive peanut monoculturing. Agronomy Journal. 2015;107(1):195.

30.	Ogundele RA, Oyedele DJ, Adekunle OK. Management of *Meloidogyne incognita* and other phytonematodes infecting *Amaranthus cruentus* and *Telfairia occidentalis* with African marigold (*Tagetes erecta*) and Siam weed (*Chromolaena odorata*). Australasian Plant Pathol. 2016;45(5):537–45.

31.	Schomaker CH, Been TH. A Model for infestation foci of potato cyst nematodes *Globodera rostochiensis* and *G. pallida*. Phytopathology. 1999;89(7):583–90.

32.	King BA, Taberna JP. Site-Specific Management of *Meloidogyne chitwoodi* in Idaho potatoes using 1,3-dichloropropene; approach, experiences, and economics. J Nematol. 2013;45(3):202–13.

33.	Garcia N, Folcher L, Biju-Duval L, Maupetit A, Ricci B, Grenier E. Impact of agricultural practices and environmental variables on plant-parasitic nematode communities in fields at a landscape scale. Nematology. 2018;20(3):211–33.

34.	Zheng GD, Shi LB, Wu HY, Peng DL. Nematode communities in continuous tomato-cropping field soil infested by root-knot nematodes. Acta Agriculturae Scandinavica, Section B — Soil & Plant Science. 2012;62(3):216–23.

35.	Zhang Z, Zhang X, Jhao J, Zhang X, Liang W. Tillage and rotation effects on community composition and metabolic footprints of soil nematodes in a black soil. European Journal of Soil Biology. 2015;66:40–8.

36.	Quist CW, Schrama M, de Haan JJ, Smant G, Bakker J, van der Putten WH, et al. Organic farming practices result in compositional shifts in nematode communities that exceed crop-related changes. Applied Soil Ecology. 2016;98:254–60.

37.	Karuri HW, Olago D, Neilson R, Njeri E, Opere A, Ndegwa P. Plant parasitic nematode assemblages associated with sweet potato in Kenya and their relationship with environmental variables. Trop plant pathol. 2017;42(1):1–12.

38.	Bongers T. The maturity index: an ecological measure of environmental disturbance based on nematode species composition. Oecologia. 1990;83(1):14–9.

39.	Bongers T, Ferris H. Nematode community structure as a bioindicator in environmental monitoring. Trends in Ecology & Evolution. 1999;14(6):224–8.

40.	Yeates GW, Bongers T. Nematode diversity in agroecosystems. Agriculture, Ecosystems & Environment. 1999;74(1–3):113–35.

41.	Whitehead AG, Hooper DJ. Needle nematodes (*Longidorus* spp.) and stubby-root nematodes (*Trichodorus* spp.) harmful to sugar beet and other field crops in England. Annals of Applied Biology. 1970;65(3):339–50.

42.	Winfield AL, Cooke DA. The Ecology of *Trichodorus*. In: Lamberti F, Taylor CE, Seinhorst JW, editors. Nematode vectors of plant viruses. Boston, MA: Springer US; 1975: p. 309–41.

43.	Norton DC. Abiotic soil factors and plant-parasitic nematode communities. J Nematol. 1989;21(3):299–307.

44.	Wah Chu K, Chow KL. Synergistic toxicity of multiple heavy metals is revealed by a biological assay using a nematode and its transgenic derivative. Aquatic Toxicology. 2002;61(1):53–64.

45.	Yu Z, Chen X, Zhang J, Wang R, Yin D. Transgenerational effects of heavy metals on L3 larva of *Caenorhabditis elegans* with greater behavior and growth inhibitions in the progeny. Ecotoxicology and Environmental Safety. 2013;88:178–84.

46.	Georgieva SS, McGrath SP, Hooper DJ, Chambers BS. Nematode communities under stress: the long-term effects of heavy metals in soil treated with sewage sludge. Applied Soil Ecology. 2002;20(1):27–42.

47.	Zhao J, Neher DA. Soil nematode genera that predict specific types of disturbance. Applied Soil Ecology. 2013;64:135–41.

48.	Rout G, Das P. Effect of metal toxicity on plant growth and metabolism: I. zinc. Agronomie. 2003;23(1):3–11.

49.	van Diepeningen AD, de Vos OJ, Korthals GW, van Bruggen AHC. Effects of organic versus conventional management on chemical and biological parameters in agricultural soils. Applied Soil Ecology. 2006;31(1):120–35.

50.	Saxton KE, Rawls WJ. Soil water characteristic estimates by texture and organic matter for hydrologic solutions. Soil Science Society of America Journal. 2006;70(5):1569–78.

51.	Prot J-C, Van Gundy SD. Effect of soil texture and the clay component on migration of *Meloidogyne incognita* second-stage juveniles. J Nematol. 1981;13(2):213–7.

52.	Nielsen UN, Ayres E, Wall DH, Li G, Bardgett RD, Wu T, et al. Global-scale patterns of assemblage structure of soil nematodes in relation to climate and ecosystem properties. Global Ecology and Biogeography. 2014;23(9):968–78.

53.	Peel MC, Finlayson BL, Mcmahon TA. Updated world map of the Köppen-Geiger climate classification. Hydrology and Earth System Sciences Discussions. 2007;4(2):439–73.

54.	Landesman WJ, Treonis AM, Dighton J. Effects of a one-year rainfall manipulation on soil nematode abundances and community composition. Pedobiologia. 2011;54(2):87–91.

55.	McSorley R. Relationship of crop and rainfall to soil nematode community structure in perennial agroecosystems. Applied Soil Ecology. 1997;6(2):147–59.

56.	Ou W, Liang W, Jiang Y, Li Q, Wen D. Vertical distribution of soil nematodes under different land use types in an aquic brown soil. Pedobiologia. 2005;49(2):139–48.

57.	McSorley R, Dickson DW. Vertical distribution of plant-parasitic nematodes in sandy soil under maize. Plant Soil. 1990;123(1):95–100.

58.	Stirling GR. Biological control of plant-parasitic nematodes: soil ecosystem management in sustainable agriculture. 2014

59.	Kumar U, Berliner J, Adak T, Rath PC, Dey A, Pokhare SS, et al. Non-target effect of continuous application of chlorpyrifos on soil microbes, nematodes and its persistence under sub-humid tropical rice-rice cropping system. Ecotoxicology and Environmental Safety. 2017;135:225–35.

60.	McSorley R, Gallaher RN. Effect of crop rotation and tillage on nematode densities in tropical corn. J Nematol. 1993;25(4S):814–9.

61.	Rahman L, Chan KY, Heenan DP. Impact of tillage, stubble management and crop rotation on nematode populations in a long-term field experiment. Soil and Tillage Research. 2007;95(1–2):110–9.

62.	Leslie AW, Wang K-H, Meyer SLF, Marahatta S, Hooks CRR. Influence of cover crops on arthropods, free-living nematodes, and yield in a succeeding no-till soybean crop. Applied Soil Ecology. 2017;117:21–31.

63.	Salminen J, Eriksson I, Haimi J. Effects of terbuthylazine on soil fauna and decomposition processes. Ecotoxicology and environmental safety. 1996;34(2):184–9.

64.	Sánchez-Moreno S, Ferris H. Suppressive service of the soil food web: effects of environmental management. Agriculture, Ecosystems & Environment. 2007;119(1):75–87.

65.	Sturz, Kimpinski. Effects of fosthiazate and aldicarb on populations of plant-growth-promoting bacteria, root-lesion nematodes and bacteria-feeding nematodes in the root zone of potatoes. Plant Pathology. 1999;48(1):26–32.

66.	Oka Y, Koltai H, Bar-Eyal M, Mor M, Sharon E, Chet I, et al. New strategies for the control of plant-parasitic nematodes. Pest Manag Sci. 2000;56(11):983–8.

67.	Wei C, Zheng H, Li Q, Lü X, Yu Q, Zhang H, et al. Nitrogen addition regulates soil nematode community composition through ammonium suppression. PLoS ONE. 2012;7(8):e43384.

68.	France weather history [Internet]. Terre-net. Available from: https://www.terre-net.fr

69.	Geosol [Internet]. Available from: https://webapps.gissol.fr/geosol/

70.	Siddiqi MR. Tylenchida: parasites of plants and insects. CABI; 2000. 856 p.

71.	Castillo P, Vovlas N. Pratylenchus (Nematoda: Pratylenchidae): diagnosis, biology, pathogenicity and management. 2007

72.	Bongers T. Identification key: De Nematoden van Nederland. KNNV Bibliotheekuitgave. 1994;46.

73.	R: The R Project for Statistical Computing. Available from: https://www.r-project.org/

74.	Oksanen J, Blanchet FG, Kindt R, Legendre P, Minchin PR, O’hara RB, et al. Package ‘vegan.’ Community ecology package. 2013;2(9). Available from: http://cran.ism.ac.jp/web/packages/vegan/vegan.pdf

75.	Legendre P, Gallagher ED. Ecologically meaningful transformations for ordination of species data. Oecologia. 2001;129(2):271–80.

76.	Legendre P, Legendre LFJ. Numerical Ecology. Elsevier; 2012. 1007 p.

77.	Rao CR. A review of canonical coordinates and an alternative to correspondence analysis using Hellinger distance. Qüestiió. 1995;19(1-2–3):23–63.

78.	Lê S, Josse J, Husson F. FactoMineR: an R package for multivariate analysis. Journal of Statistical Software. 2008;25(1). Available from: http://www.jstatsoft.org/v25/i01/

79.	Burnham KP, Anderson DR. Model selection and multimodel inference: a practical information-theoretic approach. 2nd ed. New York: Springer; 2002. 488 p.

80.	Grueber CE, Nakagawa S, Laws RJ, Jamieson IG. Multimodel inference in ecology and evolution: challenges and solutions: multimodel inference. Journal of Evolutionary Biology. 2011;24(4):699–711.

81.	Cibois P. L’analyse des correspondances : l’indispensable retour aux données. Histoire & Mesure. 1986;1(3):239–47.

82.	Cibois P. Les pièges de l’analyse des correspondances. Histoire & mesure. 1997;299–320.

83.	Andersen PK, Borgan O, Gill RD, Keiding N. Statistical models based on counting processes. Corr. 2. print. New York, NY: Springer; 1997. 767 p. (Springer series in statistics).

84.	Bates D, Mächler M, Bolker B, Walker S. Fitting linear mixed-effects models using lme4. Journal of Statistical Software. 2015;67(1):1–48.

85.	Barton K. Package ‘MuMIn.’ Version. 2013;1:18.

86.	Gelman A. Scaling regression inputs by dividing by two standard deviations. Statist Med. 2008 Jul 10;27(15):2865–73.

87.	Gelman A, Su Y-S, Yajima M, Hill J, Pittau MG, Kerman J, et al. Package ‘arm.’ 2016; Available from: ftp://apache.cs.uu.nl/mirror/CTAN/macros/latex/contrib/business-research/doc/~/mirror/CRAN/web/packages/arm/arm.pdf

**Supporting information captions**

**Fig S1 (FigS1_RDA.pdf): Outputs of the tb-RDAs for each year period of cultivation practices**

Arrows and bold texts indicate the significant variables after a permutation test. Modalities of soils depend on the proportion of sand, silt and clay in it (SoilSaSiC = majority of sand, then silt then clay; SoilSiCSa = majority of silt, than clay, then sand; SoilSiSaC = majority of silt, then sand, then clay)A: cultivation practices of year n and n-1 are implemented, the first two axes presented explain 38% of the dataset variance; B: cultivation practices of the n to n-2 are implemented, the first two axes presented explain 36% of the dataset variance; C: cultivation practices of year n to n-3 are implemented, the first two axes presented explain 35% of the dataset variance; D: cultivation practices of year n to n-4 are implemented, the first two axes presented explain 39% of the dataset variance; E: cultivation practices of year n to n-5 are implemented, the first two axes presented explain 36% of the dataset variance.

**Fig. S2 (FigS2_MCA.pdf): Outputs of the MCAs for each year period of cultivation practices.** Only the most contributing variables are projected on the maps. PPNs do not contribute to the factorial map construction. Classes for each variable are based on first quartile, median and third quartile. Abbreviations are presented in the supplementary material TableS2. A: only cultivation practices of year n (year of sampling) are implemented in the analysis; B: cultivation practices of year n and n-1 are implemented; C: cultivation practices of year n to n-2 are implemented; D: cultivation practices of year n to n-3 are implemented; E: cultivation practices of year n to n-4 are implemented; F: cultivation practices of year n to n-5 are implemented.

**TableS1 (TableS1_GLMMcomplete.xlsx): Raw model-averaging results for each taxon and year periods.** For each taxon, variable and year period considered, estimate, after the model averaging approach, 95% confident interval (95% CI) and the sum of weight (Σωi) are presented for the variables retain after the selection step.

**TableS2 (TableS2_MCAabbreviations.xlsx): Detail of the abbreviations used in MCA analysis outputs**

**TableS3 (TableS3_CompleteDataset.xlsx): Complete dataset (PPN community and environmental and cultural variables) for each year of sampling.**
