# Supplementary material for: Diversity of plant parasitic nematodes characterized from fields of the French national monitoring programme for the Columbia root-knot nematode
Source: PLoS One. 2022 Mar 8;17(3):e0265070. doi: 10.1371/journal.pone.0265070 (PMC8903304; doi:10.1371/journal.pone.0265070)
Supplement: Attachment — Submitted filename: ResponseToReviewers.docx [file pone.0265070.s007.docx]

**Response to the points raised by the academic editor and reviewers**

Dear editor,

Please find here a response to reviewers’ comments and a substantially revised manuscript according to all the points raised. According to numerous comments from both reviewers, many sentences have been modified to remove subjective language, and clarifications have been made for table 1 and Methods section. As reviewers pointed out some misuse of different terms, supporting informations have also been modified to match the vocabulary in the manuscript.

Sincerely

Dr. Nathan Garcia

**Editor comments:**

- Please ensure that your manuscript meets PLOS ONE's style requirements, including those for file naming.

_ Style and format have been checked and modified through the whole manuscript to meet PlosOne’s style requirements according to the templates from the links. Fig 1 has been uploaded to the PACE digital diagnostic tool as recommended.

- Please clarify whether this [conference proceeding or publication] was peer-reviewed and formally published. If this work was previously peer-reviewed and published, in the cover letter please provide the reason that this work does not constitute dual publication and should be included in the current manuscript.

_ This work was not peer-reviewed nor formally published. In fact, some of the results were obtained and presented in my PhD thesis, defended at the end of 2017, and the whole thesis manuscript is available on the HAL open archive (which is mandatory for French Ph.D. students), without any copyrights. Co-authors and I have continued working on this study since my PhD defense and thus the analyses and the results presented in this manuscript are not strictly the same as those from my Ph.D. thesis manuscript and most of the text has been rewritten. There is no reason to consider this submission as a dual publication of my PhD thesis manuscript.

**Reviewer #1 comments:**

- Nematodes are not microorganisms, they belong to the mesofauna, please correct

_ Reviewer #2 also raised this point. The sentence has been removed from the abstract and corrected latter in the manuscript.

- To compete is not enough, they need to suppress the non-native species. Rephrase.

_ Ideally reviewer #1 is right, however we have few evidences of such suppression as in Garcia et al., 2018 (DOI: 10.1111/ppa.12914) we observed suppression of *M. chitwoodi* in only 5 pots out of 80 after a 4 months experiment. But abundance decrease has been observed much more frequently and we can expect that over a longer period this can lead to suppression or at least keep the non-native species at a non-damageable level. So to suppress is indeed the best solution but to compete is already a quite interesting observation as it can lead to suppression or some control over time.

- “…using non-damaging indigenous species…” applies artificial application of those specimen, which is not realistic. I think what is meant is, that non-damaging species should be supported in order to suppress damaging species.

_ It is indeed what we meant. The sentence has been modified accordingly.

- Regarding the target organisms, I don’t think we can differ between non-native and native damaging species. Here we need a more general approach like “suppressing damaging species including non-native species.” Or similar.

_ We agree if the non-native species is already established in the soil. However, we placed this work in a scheme of an introduction of a species listed as quarantine organism that is not currently widely distributed in Europe and thus we consider that supporting indigenous non-damaging species may decrease the abundance of such introduced species.

- Looking of the spectrum of identified genera, they belong to more than one family. Rephrase accordingly.

_ Sentence has been rephrased. We identified 10 plant-parasitic nematode taxa, and for one of them, we were not able to identify the genus but only the family.

- Soils are part of fields. No need to mention separately.

_ Sentence has been modified. By “field” we meant “cultural practices” and by “soil” we meant “soil physico-chemical properties”.

- I don’t think data were not collected over six years, but instead field data representing the past six years were collected. Clarify.

_ Sentence has been modified. Indeed, we meant “representing the past six years”.

- … than what? Second partner of comparison needs to be mentioned.

_Sentence has been completed.

- Both aspects were not part of the study. Food quality refers to crop plant species grown and soil food web to the entire soil ecosystem. The presented data do not provide any information on the food quality of the crops grown nor on the characterization of the soil food web (suppressiveness etc). Change accordingly.

_ Sentence has been removed. We agree that the sentence was too general compared to the data analyzed in this work.

- Uncommen wording within this context. Do you mean establishment or spreading? Why managing and not controlling/eradicating/suppressing the introduced nematode? Rephrase accordingly.

_ “implantation” has been replaced by “establishment” all over the manuscript. This point was also raised by reviewer #2. To our opinion, “manage” is synonym to “controlling” suggested by reviewer #1 and as mentioned in a previous comment, we have only few evidence that “eradicating/suppressing” is possible, while abundance decrease is more obvious and already a useful lever.

- Need to be mentioned, how this can be done. This is the most relevant aspect according to the authors, and thus the procedure should be described in detail. What are the options to promote diversity? How does the information gained from the collected data specifically help to promote diversity. Give at least suggestions.

_ Sentence has been removed since the reasoning was too indirect for the abstract for which we do not have space for more details. Here we meant that the variables highlighted in our work could positively influence PPN communities’ richness and abundance and thus promote biodiversity.

- See previous comment

_ “micro-organisms” replaced by “mesofauna”.

- No need to mention

_ Agree, text has been removed.

- What are natural population levels? Needs explanation.

_ Reviewer #2 also raised this point. “population level” has been modified by “abundance” all over the manuscript. By “natural abundance” we meant the abundance that is commonly observed in French fields, both according to our expertise but also to published data like Villenave et al. (2013) (DOI: 10.4236/ojss.2013.31005) in which they sampled and analyzed the nematode communities from 52 fields or Garcia et al. (2018) (DOI: 10.1163/15685411-00003136) where 72 fields were considered.

- establishment?

_ Yes, word has been replaced.

- See previous comment

_ Same.

- What do you mean by “competition between communities”? specify.

_ Sentence has been rephrased.

- I guess it is more the composition than the structure

_ As reviewer #2 suggested, we have replaced “structuration” by “community structure” for the whole manuscript.

- It is not clear to the reader how that information will improve national monitoring programs. A good understanding of the diversity and composition of nematode communities is highly complex and time consuming. I doubt that it will be realistic in the future to select fields in monitoring programs based on nematode diversity and composition. The main and most likely only factor is host plant status. Rephrase accordingly.
- As mentioned above, if you want to find *M. chitwoodi*, you sample good host plants at the end of the season (highest expected numbers) considering possible ways of entrance (e.g. fields were seed potatoes were planted). Composition of plant-parasitic nematodes is not an issue and I doubt it will be in the future.

_ Sentences pointed out by these two comments has been modified to make our reasoning clear. We totally agree that for national monitoring program for *M. chitwoodi*, it is essential to sample fields based on good host plants and we are not questioning this in the study. However, it is not humanly possible for the sanitary services to sample every fields with a good host plant in the country. Thus, we wanted with this work to promote additional elements that can be considered to choose in the most efficient way the fields to sample among those with a good host plant. As indigenous plant-parasitic nematode communities may decrease *M. chitwoodi* abundance, and as soil properties, cultural practices and climate may impact plant-parasitic nematode communities, we believe those should be relevant elements to consider in addition to the host plant.

- Tillage per se can also enhance plant-parasitic nematode densities, such as minimum tillage. Thus it is more the intensive tillage including ploughing that has a negative impact on ppn. Please specify.

_ Sentence has been modified.

- “soil” indicates all nematodes, but non-plant parasitic nematodes are not covered in this study

_ Agree, the modification proposed by reviewer #1 has been accepted.

- Those numbers are quite high. Is this really juveniles or could it be cyst content (eggs + juveniles)?

_ It is an extrapolation of Heterodera cyst content.

More details have been added in Methods section according to a latter comment from reviewer #1 and a comment from reviewer #2

In a previous work published by Garcia et al. 2018 in Nematology (DOI 10.1163/15685411-00003136) we investigated the number of juveniles and eggs in Heterodera cysts sampled in 7 fields in Burgundy (France) during two consecutive years. A total of 33 cysts belonging to different species were individually crushed and the number of juveniles and eggs counted. The obtained mean juveniles + eggs per cyst was then used to convert cyst numbers of other French fields into juvenile and eggs numbers.

- See comment above

_ We removed “micro” as suggested

- composition?

_ “Structuration” has been replaced by “community structure” as suggested by reviewer #2

- Composition?

_ See previous comments.

- It is not obvious how this can be the case and thus needs explanation.

_ Sentence has been more detailed. We meant here that profound tillage modifies soil texture, destroy weeds and move the nematodes deeply in the soil. Since these tiny organisms have limited active migration abilities, profound tillage negatively affects food accessibility.

- Composition?

_ See previous comments.

- What kind of groups? Needs explanation

_ We detailed here the group of years mentioned in Table 2.

- Or alternatively, ….were lowest in soil with the lowest C/N ratio.

_ The suggested changes have been accepted.

- Pesticides in general? The survey only mentions herbicide. Stick to what has been studied and avoid generalizing things.

_ Sentence containing this term has been removed (see following comments from reviewer #2). However, to answer the question, here we meant pesticides in general (including herbicides). In the survey, farmers have indicated the number of herbicide application and other pesticides application (meaning fungicides, molluscicides and insecticides) that we called “non-herbicides” in the manuscript. As farmers are not always prone to give details about the other pesticides applications, we have had to stick to the sole total amount of non-herbicides application.

- are soil work, tillage and cultivation practices used synomously? If so, stick to one term (cultivation practices) and use this consistently. Change in entire text.

_ In the manuscript “cultivation practices” is more general and contain tillage practices, herbicide and non-herbicide applications and crop rotations.

Furthermore, according to reviewer #2 comment, “soil work” have been replace by “tillage practices” in the entire manuscript and we have detailed “superficial” or “profound” each time.

- Mention the criteria for the selection of those fields

_ Details about the selection of the fields in the French monitoring program have been added.

- Too general as not all heavy metals were determined. Specifically mention those that have been considered in this study.

_ “Heavy metals” have been replaced by those considered in this work.

- I don’t think there are techniques that distinguish between free-living and cyst nematodes. But there are techniques specifically for mobile stages and cyst stages. Is that what is meant? Needs clarification and precise wording.

_ Yes, we meant “mobile stages”, “free-living” has been replaced.

- Using the same technique, i.e. Oostenbrink elutriator? This does not make sense. Describe more preciously how extraction was done and mention a reference

_ The whole extraction paragraph has been substantially more detailed, according to the appropriate reference, the EPPO extraction protocols for nematodes.

- Two times the same centrifugation? Needs explanation.

_ More details have been added. The first centrifugation is done with water and the second with MgSO_4_ with a density of 1.18.

- See previous comment

_ “free-living” has been removed from the modified sentence.

- I thought the nematodes are already extracted at this step. So, where does the soil extract comes from? Do you mean nematode suspension? Usually, nematodes are concentrated in a certain volume (e.g. 10 ml) and then an aliquot (e.g. 1 ml) is used to identify and count the specimen. Preciously describe the method used.

_ Yes we meant “nematode suspension”, the words have been replaced. More details about the dilution step have also been added. We counted the PPNs from 5mL of a diluted suspension (usually 100mL) depending of the abundance of nematode in the total suspension to be able to correctly identify and count them.

- …. or eggs plus juveniles per cyst? (= cyst content)

_ Yes we meant juveniles and eggs. Sentence has been modified accordingly.

- Difficult to understand how this was done. Generally, a defined number of cysts is crushed and cyst content in terms of number of eggs and juveniles determined and calculated back according to 100 g soil.

_ See previous comments for the detailed method. Information has been added.

- What is the message of Legendre and Gallagher? Explain

_ Details about the cited reference have been added at the end of the sentence.

- The number of references is far too long for a research article of this type. Keep in mind that this is not a review article. Condense number of references to 30-40. This can be easily done by focusing on the most relevant references and/or referring to review articles regarding general aspects.

_ We have reduced the number of cited reference by 13 but we preferred and strongly believe that it is best practice to refer to original publications than to reviews citing these original publications. Moreover, PlosOne guide for author does not limit the number of references.

**Reviewer #2 comments:**

- Line 44: Technically unicellular organisms are considered to be micro-organisms, whereas nematodes (multicellular) are considered microfauna or mesofauna. Please adjust this wording for clarity and consistency with the field.

_ See comment from reviewer #1.

- Line 47: Does “natural population levels” refer to crop population abundances or nematode abundances? Please specify.

_ “Population level” has been replaced by “abundance” in the whole manuscript according to reviewer comment.

- Lines 55-57: “…could be potential antagonists.” This sentence is unclear, and the implication is vague; please specify what the introduced species could be antagonists for or to, and how the antagonism might occur.

_ Sentence has been rephrased and an example of antagonism between indigenous PPN communities and introduced species is given in the following sentence.

- Line 70: The term “soil work” is a non-standard term that is used frequently throughout this manuscript in reference to cultivation practices. In the interests of clarity, please use the standard terms “tillage practices” or “cultivation practices”, and further detail as necessary.

_ “Soil work” has been modified in the manuscript and figures accordingly to this comment.

- Line 72: Please describe how cultivation and physico-chemical properties of the soil affects nematodes. When creating a line of reasoning it is not sufficient to simply state that there is an effect, the effect should be described.

_ Details have been added to the sentence.

- Line 73: Please list types of environmental conditions and crop practices that have been studied.

_ Sentence has been completed.

- Lines 73-74: Please describe the nature of restricted areas and limited experimental designs.

_ Sentence has been removed to avoid repetition with the previous and following ones.

- Line 78: Please remind us of the importance of a root crop vs. a cereal crop.

_ Details have been added and the beginning of the Methods section has also been more detailed according to a latter point raised by reviewer #2.

- Lines 104, 106: Please specify whether per 100 g soil refers to 100 g wet (or field-moist) soil or dry soil?

_ We meant 100g of fresh soil. This information has been added.

- Lines 107-109: I did not see any information in the Methods about performing calculations for the Shannon Index. Please include methodology, reference(s), and data.

_ Those informations have been added to the Methods section. A line regarding Shannon index has been added to table 1.

- Lines 110-112. This sentence is unclear. What does this mean?: “…and Meloidogyne sp. mentioned in the article correspond to other species.”

_ In French legislation, it is mandatory to look for *Meloidogyne chitwoodi* and *M. fallax* if we found any *Meloidogyne sp.* individuals in a sample. As we found some *Meloidogyne* in several samples, we ensured that it was not one of the two quarantine species. Sentence has been modified to make it clear.

- Line 136: Misspelling of “this”. “…cumulative effect of few practices…”; please list the practices to which you refer. Also, it is mentioned that agricultural practices were not retained in the analysis, and the sentence immediately contradicts itself by stating that effects due to practices were observed. Could this entire sentence be clarified?

_ Sentence has been clarified. On Fig 1, only cultural practices of the sampling year are considered and none significantly affects the PPN communities (*i.e.* none appeared on the factorial map). However, when the cultural practices from the past years are also considered, effect of the successive non-herbicide applications and of the successive profound tillage appeared on the factorial maps presented in Fig S1.

- Line 138: Please specify what you mean by a field surface. Whether or not a result is difficult to explain it should be listed in the results; further explanation can be done in the discussion, and if indeed the explanation is due to analysis artifacts, further explanation of the nature of the artifact is needed.

_ Field surface is the field area, measured in hectare.

This result is still present in the Results section but we move the rest of the paragraph regarding a possible artefact in the Discussion section with clarifications of our reasoning (line 271-277 in the track changes version).

- Lines 139-140: The methodology as described in this sentence appears to match sampling protocols outlined in the Methods section, thus contradicting the opening of the sentence. Please describe in detail what was not adaptable for this particular sampling.

_ Here we meant that we did not sample more soil in largest fields. Sentence has been modified to clarify.

- Line 143: Please reference the appropriate figure or table after “…factorial map.”

_ Reference to the Fig 1 has been added.

- Line 144: “seem” is subjective; simply list the impact of the variables quantitatively or statistically rather than rendering a judgment call as to perceived importance. Commentary of that nature should be moved to the discussion.

_ Sentence has been modified to remove “seem to”.

- Line 144: By “structuration” do you mean “community structure”? Structuration is not the standardly accepted term for community structure, nor is it commonly used to refer to the formation or change of community structure.

_ Yes, we meant “community structure”. The word has been modified for the whole manuscript.

- Lines 167-182: The definitive statements “None of these variables impacted…”, “…had a positive impact…”, “…had a negative impact…” cannot be made. Remember that this study is largely statistical, and that that metadata originates from soil data that has not been measured in the laboratory for this study. At most it is possible to state that “…have appeared to positively/negatively impact…”. This paragraph requires significant clarification.

_ The whole paragraph has been modified to remove definitive statements about soil variables (lines 183-200 of the track changes version). We agree that the soil data were not measured during this work but we have minimized this bias as much as possible by using the database developed by the French National Research Institute for Agriculture, Food, and Environment. Data are available at the canton scale (the smallest French administrative scale with a mean surface of 192Km²). For each canton, more than 10 soil samples were analyzed over the canton surface between 2010 and 2014. As the samples for this work took place in 2015 and 2016, we considered that those data were close enough to our conditions.

- Lines 187-188: “Various” is vague usage. Please specify the geographical areas and crop management approaches to which you refer.

_ Sentence has been clarified. This first sentence was general to highlight that each field presented different environmental conditions (climatic and soil) and different cultural practices (for example from 0 to 6 profound tillage for the past 6 years).

- Line 189: What do you mean by “close to that of other similar agrosystems”? “Close” is subjective; what of your data supports this assertion, and please be very specific in your response. What are “similar” agrosystems?

_ Sentence has been clarified. We meant agrosystems that also include cereals and vegetables in the crop rotation similarly to the fields sampled in our study.

- Lines 194-195: “Majority of taxa” is undefined. Please specify percent or number of taxa. “Several regions” is undefined. Specify how many regions, which ones they are, and discuss the importance of these regions with respect to crops, nematodes, etc. “Seems to vary” is undefined and subjective. Specify numerically or statistically the degree to which taxa vary, and what they vary with respect to (presence, abundance, etc).

_ Sentence has been modified to give numbers of taxa and regions. We remove subjective end of the sentence, which is discussed in the following sentence.

- Line 203: Please specify what economic issues are induced.

_ We specify the economic issues from the cited references.

- Line 204: Rather than “One of these two crops…”, crops should be specified.

_ Crops (beet or carrot) mentioned in the previous sentence have been specified here.

- Line 214: This entire sentence is vague. Specify soil conditions to which you refer, and how these collective soil conditions and/or abiotic factors influence PPN and free-living nematodes. Discuss how these collective soil and abiotic factors stimulate or repress nematode growth and development, and why this is important to your argument.

_ Sentence has been modified to be more specific about the abiotic factors and the discussion about their impact is detailed during the rest of the paragraph.

- Line 216: “More or less susceptible” is vague. Explain how nematodes are susceptible to the presence of Zn and Cu in (presumably) soil.

_ Sentence have been detailed with information from the cited reference.

- Line 220: Do you mean “intoxicated” or nematodes that suffer from toxic effects?

_ Yes we meant “intoxicated”. Sentence has been slightly modified to make it more understandable.

- Lines 222-223: Specify the “strong impact” due to heavy metals.

_ Sentence has been clarified in order to develop the reasoning.

- Line 228: “Major” is subjective; simply list the impact of soil texture on community structure quantitatively or statistically rather than rendering a judgment call as to perceived importance. Is the effect of statistical significance? Usage of “structuration” again.

_ We removed the word “major” and added information about the kind of impact of soil texture. Furthermore, more details are given in the following sentences.

- Line 231: “Eased” is subjective. Please clarify.

_ Sentence has been clarified to explain that root exudate are used by nematodes to locate their host plants.

- Lines 235-237: Specify how rainfall affects nematode communities, how temperature affects PPNs, and why other soil variables are not considered important (and specify the soil variables). Why are all of these effects the case?

_ These sentences have been reformulated and more details given about the effects of rainfalls and temperature observed in the cited reference.

- Line 239: Sentence appears to contradict itself, can this be made clearer?

_ Sentence has been clarified. Here we meant that the French climate is similar from north to south regarding the Köppen-Geiger climate classification map.

- Lines 242-243: “Soil work” and “structuration”—see notes above.

_ See previous comments.

- Lines 253-260: I’m having trouble following the flow of the argument; please clarify. Also, on line 256, the statement is made that the study has a large geographic scale, whereas in line 239 the opposite is stated. Please clarify.

_ Paragraph has been detailed to clarify our reasoning. Concerning the geographic scale, we meant here that at the country scale of this work, environmental variables have more impact on communities’ structure than the crop rotations that are very similar across the sampled fields (majority of cereals). Line 239 on the contrary, we stated that France is not a large country compare to the worldwide scale studied in the cited reference (Nielsen *et al.* (2014)) to highlight that climatic variations in our work are less diverse than climatic variations at a worldwide scale.

- Line 266: “certain groups of years”. Specify the dates and/or date ranges.

_ See reviewer #1 comment.

- Line 271-272: “Their effects appeared to be positive or negative, probably depending on the PPN ecological niche.” This sentence is vague and requires considerable expansion; please discuss the specific positive and negative effects, and how these effects are or could be driven by a given ecological niche.

_ Sentence has been removed as discussion about soil physico-chemical variables impacts on PPN taxa occur in the following sentences.

- Line 273: “inputs”; please specify the inputs that you are referring to and how inputs are characterized to increase yield.

_ Sentence has been slightly modified. We meant here “fertilizers” that are used to enrich the soils for better plant growth.

- Line 274: “better quality” is subjective. What, specifically, constitutes quality, a change in quality, and what do these changes induce in nematode consortia?

_ “better quality” has been removed and we have detailed what we meant here. Positive effect of fertilization on PPN abundance may be due to more dense root systems that will allow a higher PPN development as the resource is abundant.

- Lines 285, 287: In the discussion crop rotations are described as having been removed from analyses, therefore it should not be possible to draw conclusions from them. Please clarify.

_ First sentence of the Conclusions section has been removed as it is now more detailed in the discussion about the crop rotations (see previous comments).

- Line 289: By “population” do you mean “abundance”?

_ Yes. The word “population” has been consequently modified by “abundance”.

- Lines 317-318, 320: It is safe to say that many readers will not be familiar with the national monitoring program. Within the regions listed, please specify field site locations, including the geolocations of the sites. In line 320, specify which regions, sites, and fields were used and note where changes occurred between years.

_ According to reviewer #1 comment, details about the French monitoring program for *M. chitwoodi* and *M. fallax* have been added.

It is not possible to give more details about the geolocation of the fields as this information is confidential for the French phytosanitary services and farmers agreed to participate to this work only on an anonymously basis.

As requested, informations were added to highlight where changes occurred between years (lines 366-367 of the track change version).

- Line 322: Specify the name of the agricultural data website, not just the reference. Were mean temperatures obtained for each field individually? If not, how were temperatures determined?

_ We specified the name of the website and gave more details about the data collections. Data were collected for each field individually from the closest meteorological station.

- Line 333: What is an “elementary soil core”? This is not terminology that I am familiar with.

_ In this work, it is a soil carrot over the first 30 centimeters of soil.

- Line 344: Should this be “sieves”? If so, please specify all sieve sizes as well as any standard protocols used.

_ More information has been added about the sieves and the whole extraction processes, following other comments from reviewer #1

- Line 345-346: “based on personal observations” is vague; specify the exact methodology or calculation required to determine the number of larvae per cyst.

_ In a previous work published by Garcia et al. 2018 in Nematology (DOI 10.1163/15685411-00003136) we investigated the number of juveniles and eggs in Heterodera cysts sampled in 7 fields in Burgundy during two consecutive years. A total of 33 cysts belonging to different species were individually crushed and the number of juveniles and eggs counted. The obtained mean juveniles + eggs per cyst was then used to convert cyst numbers of other French fields into juvenile and eggs numbers.

- Line 351: Please define “field surface”.

_ See previous comment.
