# Supplementary material for: Diversity of plant parasitic nematodes characterized from fields of the French national monitoring programme for the Columbia root-knot nematode
Source: PLoS One. 2022 Mar 8;17(3):e0265070. doi: 10.1371/journal.pone.0265070 (PMC8903304; doi:10.1371/journal.pone.0265070)
Supplement: Attachment — Submitted filename: ResponseToReviewers.docx [file pone.0265070.s008.docx]

**Response to the points raised by the academic editor and reviewers**

Dear editor,

Please find here a response to reviewer 3 and your comments and a revised manuscript according to all the points raised. All the comments have been addressed except the inclusion of metadata regarding PPN communities in fields where *M. chitwoodi* is present (see following comment) as we did not find such relevant information in the scientific literature. However, we have slightly modified the text to consider those comments.

Sincerely

Dr. Nathan Garcia

**Editor comments:**

- I agree with Reviewer 3 that the inclusion of supporting metadata from fields that are indeed infested with M. chitwoodi could potentially strengthen the discussion and value of your results. I acknowledge that your study was initially designed to identify parameters that could aid the development of a focused M. chitwoodi monitoring scheme, but as you (fortunately for the owners of the sampled fields!) did not detect the species, you could consider putting less emphasis on this objective. As we are far from understanding which parameters/variables are key to the distribution of nematode taxa in general, the results on the plant parasitic nematode community is valuable on its own, even though you did not find M. chitwoodi.

_ We agree that metadata about nematode communities in fields infested with *M. chitwoodi* would be an interesting implementation in this study. However, we did not find any relevant publication in the literature to do so since we only found publications focusing on experimental design instead of agricultural fields. That appear not suitable for comparison with our study since the experiments in those studies had a major impact on the nematode communities and *M. chitwoodi* abundance prior to any other parameter. Furthermore as *M. chitwoodi* is regulated in many countries, fields that are known to be infested are usually under specific management that depend on the countries legislation. These managements implies that the whole nematode communities are drastically impacted as there is no specific management targeting *M. chitwoodi* alone. Thus, to our opinion, even those data would not be very relevant to compare with our work because they would not displayed the nematode communities commonly found in agricultural soil as we are doing in this study.

However, we have followed your suggestion to put less emphasis on *M. chitwoodi* in the revised version of the manuscript as we totally agree that impact of the studied variables on PPN communities is valuable on its own. We still believe that this kind of study can help the phytosanitary services to target the fields in their monitoring scheme as we know that PPN communities have an impact on *M. chitwoodi* abundance (since we observed it in Garcia et al. 2018 (DOI: 10.1111/ppa.12914)). But as you mentioned, we are far from understanding the whole mechanisms of soil nematode communities distribution. Thus several sentences of the abstract and introduction have been removed or modified.

- L. 29: ”anthropic … variables” sounds a bit odd; please rephrase to “cultivation practices and environmental variables”.

_ Agree, the sentence has been modified and according to the following comments, “anthropic” has been replace by the appropriate term for the whole manuscript.

- Throughout manuscript: Do not use “anthropic”, please change to “anthropogenic” or, if appropriate, use “cultivation practice”, which more accurately describes the nature of anthropogenic activities.

_ See previous comment

- L. 35: Again, there is no such thing as “anthropic soil variables”.

_ See previous comment. Here we meant “the cultivation practices that have an impact on soil” (such as tillage).

- L. 44: In soil, I would claim that nematodes belong to the microfauna (as defined as metazoan with a body width ≤100 µm). If you classify according to body length, nematodes belong to the mesofauna. However, the body width is much more relevant, as it determines which soil pore opening are accessible to the nematodes.

_ We agree but both reviewers 1 and 2 considered that nematodes belong to mesofauna. We have remove this terms from the sentence.

- L. 47: Like reviewer 1, I am also puzzled by the “natural abundances”; what would define “unnatural abundances” then? Better to state that most PPNs are not damaging to crops at the abundances commonly found in French agricultural soils (or something equivalent).

_ Agree, this is exactly what we meant here and the sentence has been modified accordingly.

- Table 1: Provide more information in the table legend; e.g. that data depict the mean of 35 and 31 fields surveyed in 2015 and 2016, respectively, and clarify what “Prevalence” and “W” refer to.

_ Table legend has been detailed accordingly.

- Fig. 1: In l. 123-125, you mention that Fig. 1 includes the cultural practices, but I only see environmental variables, no reference to cultivation practices. Please clarify.

_ Here we meant that, regardless the outcome of the factorial analysis (*i.e.* the factorial map presented in Fig.1), cultural practices were implemented to run the analysis and that for fig. 1, only the cultural practices of the sampling year were implemented (not those of the past years that are implemented to run the analyses shown in S1Fig.). As tb-RDA allow a selection step thanks to a permutation test, Fig.1 display only the significant variables which are, as you mention, only environmental variables.

The text has been modified in order to clarify.

- Table 2: Please explain the variables (C/N, Herbi, Zn) in the legend. The meaning of “Year period” should also be explained. Also describe which type of model the table depict; in essence, the table and figures should be self-explanatory.

_ Legend has been substantially detailed and the model type (*i.e.* Poisson GLMM) has been added in title.

- L. 210-211: Which better host plant?

_ It is impossible to say as species of this family feeds on a very large host range, including cereals that have mainly been grown during 2016 for the sampled fields, but also many weeds that have not been considered in this study. However, we have slightly detailed what we meant in the sentence.

- L. 215-236: You do not discuss your own results here; please do so.

_ Agree. The whole paragraph has been detailed to link the literature knowledge to our own results.

- L. 216-226: Are the referenced studies on heavy metal effects conducted in soil or in other matrices/media? Generally, the bioavailability and negative effects of heavy metals on soil organisms are dramatically reduced in soil compared to e.g. liquid media, so this information is quite important to evaluate the comparability to your study.

_ References 16, 33, 34 studied heavy metal effect on nematode communities in soil and references 31 and 32 studied their effect on *C. elegans* on a growing media but observed detrimental effect even at low concentration. In our discussion, references 16, 33 and 34 are cited to be compared to our results and while reference 31 and 32 are cited to explain the physiological mechanisms involved. As the paragraph has been modified accordingly to the previous comment, we also included these elements.

- L. 218: Please add a reference that shows negative correlation between Zn conc. and PPN abundance.

_ L.218, we were discussing about our own results. We agree that it was not clear and thus the sentence has been modified.

- L. 245: Coming from a small country, I find that France is a rather large country. In any case, the important issue here is not the size of the country, but climatic variation, so I suggest you omit reference to the size of the country and focus on climatic variation across the sampled regions.

_ Agree, and this is what we meant. Reference to the country size has been removed in the text.

- L. 249: “field surface area”.

_ Sentence has been replaced

- L.250-51: “… we did not sample more samples in the larger fields), but sampled seven soil core over the longest diagonal in all fields”

_ Sentence has been replaced

- L. 252: “surface area affected PPN communities”

_ Sentence has been replaced

- L. 269: “contrasts”

_ Corrected

- L. 368: “surface area”

_ Sentence has been modified

- L. 388: Please define “MCA”

_ MCA (for multiple correspondence analysis) has been defined

- L. 402 and 403:”surface area”

_ Sentences has been modified

- L. 405: Define “SW” and “CI”

_ SW (for sum of weight) and CI (for confident interval) have been defined.

**Reviewer #3 comments:**

- In the abstract, introduction and discussion the authors emphasize that the obtained knowledge described in this work can facilitate management of the establishment of an introduced species (M. chitwoodi). Basically the aim is to look for parameters to develop an more focussed sampling scheme to monitor M. chitwoodi/M. fallax infestations. Unfortunately in none of the sampled fields M. chitwoodi was detected. It would have been an added value if M. chitwoodi-infested fields were included in this study. If metadata are available for known infested fields it would be useful to compare these with the results from this study and add this comparison to the discussion. With the provided data no conclusion can be made regarding higher possibilities for establishment of M. chitwoodi.

_ See first comment from editor.

- Statement in line 49-51 is not backed by references given, other refs should be used

_ References used here has been modified.
